# Immunologic, virologic and drug resistance outcomes in an HIV-infected prospective cohort on treatment in South Africa

**DOI:** 10.1371/journal.pone.0307519

**Published:** 2024-08-26

**Authors:** Bixa Ogola, Nontokozo D. Matume, Denis M. Tebit, Lufuno G. Mavhandu-Ramarumo, Pascal Obong Bessong

**Affiliations:** 1 SAMRC-UNIVEN Antimicrobial Resistance and Global Health Research Unit, HIV/AIDS & Global Health Research Programme, University of Venda, Thohoyandou, South Africa; 2 Discipline of Genetics, School of Life Sciences, University of KwaZulu-Natal, Pietermaritzburg, South Africa; 3 Global Biomed Scientific LLC, Forest, VA, United States of America; 4 Center for Global Health Equity, University of Virginia, Charlottesville, Virginia, United States of America; 5 School of Health Sciences, University of KwaZulu-Natal, Durban, South Africa; Nazarbayev University School of Medicine, PAKISTAN

## Abstract

**Background:**

In September 2016, South Africa introduced the Universal Test and Treat (UTT) programme to manage HIV infection. However, the development of drug resistance and sustaining viral suppression are challenges to the success of treatment programmes. This prospective observational study describes virologic, immunologic, and drug resistance profiles in a test and treat cohort in north-eastern South Africa.

**Methods:**

Five hundred and thirty-four HIV-1 positive antiretroviral naïve adults entering treatment programmes were enrolled between January 2016 and February 2018. Trends in CD4+ cell count, viral load, and drug resistance by examination of deep sequences were assessed at baseline and every three months, for 24 months.

**Results:**

Seventy-five percent were late initiators into ART (that is baseline CD4+ cell counts < 500 cells/microliter) and 16% were early initiators into ART and baseline CD4 was not available for 9%. Eleven percent (12/104) achieved immunological response after 6 months, 39.4% (41 /104) after 12 months, and 97.5% (101/104) after 24 months. Seventy-one percent (381/534) had baseline viral loads >1000 RNA copies/ml. Nine percent (22/246) achieved viral suppression after 3 months, 50% (122/246) after 6 months and 73.6% (181/246) after 12 months. A slower viral suppression was observed for males than females (p value = 0.012). A total of 45.6% (52/114) individuals had at least one drug resistance mutation (DRM) detected at >20% threshold in any of the time points, and the number increased to 55% (63/114) when minor variants were accounted for. Forty-eight percent (14/29) had drug resistance mutations at >5% threshold as early as 3 months into treatment.

**Conclusion:**

The UNAIDS target of 95% viral suppression in individuals under treatment was not observed after 12 months of treatment, and this was less successful for males. Adherence and drug resistance monitoring could be beneficial for individuals harbouring resistant viruses early into treatment.

## Introduction

The Joint United Nations Programme on HIV/AIDS (UNAIDS) has set a 95-95-95 goal by 2030. This requires 95% of individuals to know their HIV status, 95% of infected persons to be on antiretroviral therapy (ART) and 95% of those on ART to have sustained viral suppression [1]. The goal has been widely adopted, and thus far there are two countries in Africa; Botswana and eSwatini that have achieved this goal [2,3].

South Africa, a country with an estimated 8 million HIV-infected individuals as of 2021, is still on its way to reaching the 95-95-95 goal. Thus far, 90% HIV infected persons in South Africa know their HIV status, 91% are on ART, and 94% of those on ART have sustained viral suppression [4]. To support treatment as prevention, and to meet the UNAIDS 2030 goal, the country in September 2016 introduced Universal Test and Treat (UTT) [5]. The UTT approach entails that all those diagnosed with HIV should access treatment, irrespective of previous ART initiation guidelines based on CD4+ cell counts. Early ART initiation has been shown to produce better health outcomes which includes improvements in CD4+ cell counts, and faster viral suppression compared to deferred ART initiation. Also, early ART initiation significantly increases the uptake of ART [6–8]. However, there are obstacles to the success of such treatment programs.

Expanded guidelines such as the UTT are critical to getting more patients into HIV treatment. However, universal access to treatment may not be sufficient to achieve the UNAIDS 2030 goal, if it is not accompanied by effective approaches to detect prior exposure to ART before treatment initiation, support adherence to treatment, early detection of drug resistance, and understanding of drug metabolism and transport in certain individuals [9,10]. Therefore, the current study examined the trends in CD4+ cell count, viral load, and drug resistance in a cohort of persons entering the test and treat programme in north-eastern South Africa.

## Materials and methods

### Ethics approval

The study included human participants and the protocol was approved by the Human and Clinical Trial Research Ethics Committee of the University of Venda (SMN/15/MBY/23/0710). Permission to access public sector health facilities was obtained from the Limpopo Provincial Department of Health, and the relevant district health managers. Signed written informed consent was obtained from all participants prior to enrolment in the study. Minors were not enrolled in the study. Confidentiality and anonymity were maintained throughout sample processing and data analysis.

### Study population

Consenting HIV positive drug naïve participants preparing to enroll into ART programmes at four public sector health centres were included in the study as described by [11]. However, recruitment of participants commenced from the 14^th^ of January 2016 to the 27^th^ of February 2018. These centres offer treatment for HIV, AIDS, and TB, along with social support for patients. These centres initially offered maternity services, including short-term accommodation for pregnant women and postnatal care. Inclusion criteria used in the study were that the participants were HIV positive, ART naïve and were to initiate ART at the time of recruitment into the study. Data on age, sex, marital status, highest level of education, occupation, income, probable place of infection, probable year of infection, probable risk factor for infection, starting antiretroviral (ARV) regimen, date of treatment initiation, place of residence, other diseases at initiation of HIV treatment, WHO clinical HIV disease stage, data on prior exposure to ARV was obtained by self-reporting at recruitment.

### Sample collection and processing, viral load and CD4+ cell count measurements and RT-PCR

Whole blood (3ml) was collected and processed as mentioned in [11]. It was collected into EDTA vacutainer tubes prior to treatment initiation, and every three months to sync with normal clinical visits of each of the 534 recruited participants, over a period of 24 months. Additional data such as treatment status, the wellbeing of the participants (if they are alive), and reasons for exiting the study for participants that exited were obtained from participants’ medical records as well as through follow-up phone calls. CD4+ T-cell count was performed on a BD FACSPresto (Becton Dickinson) instrument according to the manufacturer’s instructions. Viral load was done at Lancet Laboratories Pty (Ltd), Pretoria, South Africa), on a COBAS AmpliPrep/COBAS TaqMan HIV-1 Test v2.0 platform (Roche, USA).

RNA was extracted from plasma with Qiagen RNA extraction kit, according to the manufacturer’s instructions and stored at minus 80°C until use. Extracted viral RNA was reverse transcribed and amplified by nested PCR as previously described, to obtain about 1.6kb fragment comprising the entire protease and approximately 900bp of the reverse transcriptase gene [11]. Amplicons were purified using AMPrep XP beads (Beckman Coulter, USA).

For quality assurance, pilot work was done to optimize protocols and positive (mj4 plasmid) and negative controls were used for each PCR run. PCR prep and sequencing library prep were each carried out in Pre-PCR, Post-PCR and NGS sequencing rooms using biosafety level 2 protocols. Sequencing libraries were barcoded and spiked with internal control (PhiX) during library prep as mentioned in [11]. Sequence read quality control was performed as mentioned in [11].

### Definition of immunologic and virologic outcomes

A positive immunologic outcome was defined as an increase in CD4+ cells by 50 cells/μL after 6 months on ART. Participants whose baseline CD4+ cell counts were greater than 500 copies/μL were classified as early initiators into ART (EIA) and those with less than 500 copies/μL were classified as late initiators into ART (LIA). The primary measure of virologic outcome is viral suppression which was defined as a reduction in baseline viral load count to less than 50 copies/mL after 3 months of initiating ART.

### Next generation sequencing, drug resistance detection, and genotyping

Library preparation, next-generation sequencing, post-sequencing quality control, consensus sequence generation, variant calling, and drug resistance mutation interpretation were carried out as described previously by [11]. Briefly, library preparation was done using an Illumina Nextera XT DNA Sample Preparation Kit (Illumina San Diego, California, USA) and sequencing was done on an Illumina MiniSeq instrument using a Mid-output Kit for 300 cycles to generate paired-end reads (Illumina San Diego, California, USA). Sequences were paired and de-multiplexed automatically on the MiniSeq as part of the data processing steps. Sequence quality was validated using the FastQC program. The fastq files were imported into Geneious PRIME software version 2020.1.2 [12] for filtering and trimming of sequences as well as downstream analysis. A total of 3 consensus sequences were generated per sample, at >20%, >5% and >1% variant calling thresholds. Drug resistant mutation was interpreted with the web-based Calibrated Population Resistance (CPR) tool within the Stanford HIV Drug Resistance Database (https://hivdb.stanford.edu/cpr).

Drug resistance mutations (DRMs) were detected at different variant frequencies. The >20% threshold which accounted for majority variants and the >5% and >1% thresholds which accounted for minority variants were extracted using the find variation/SNPs feature from the annotation and prediction menu in Geneious. A phylogenetic tree was edited using Interactive Tree of Life (iTOL) https://itol.embl.de/.

### Statistical analysis

Statistical analysis was done using R version 4.1.0 within RStudio desktop version 1.4.1717. Descriptive statistics of the study population was done using the functions in the R base and psych package [13]. Viral suppression and immunological response were determined from Kaplan Meier curves plotted using functions in the survival and survminer packages. Associations between patient demographics and clinical data to viral suppression or immunological response were determined from Cox proportional hazard model [14]. The predictor variables were age, sex, marital status, baseline CD4+ cell count and HIV viral load. Correlations between viral loads and drug resistance mutations were investigated using the R Studio statistical program.

## Results

### Characteristics of the study cohort

**Fig 1:**
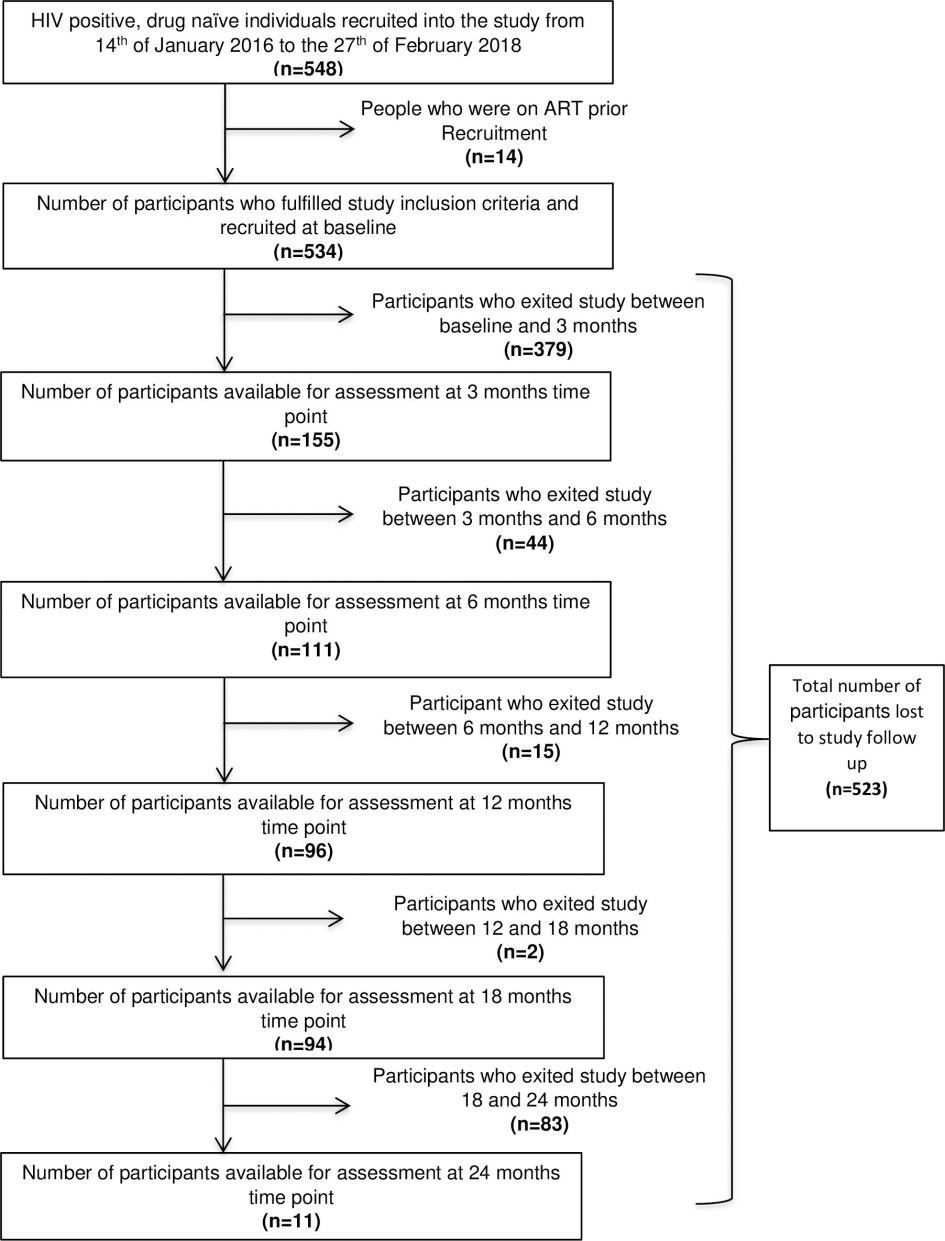
Flow diagram of individuals recruited into the study and followed for 24 months.

In total, 548 participants were recruited into the study. However, 14 were excluded from the study as they did not fit the inclusion criteria (Fig 1). The remaining 534 study participants were comprised of 378 females (71%). Sixty-five percent of the participants were single. The median age of the study population was 35 years (IQR: 29–44). A total of 30.5% (163/534) of participants reported to have likely acquired HIV-1 infection between 2002 and 2014; 35.8% (191/534) reported to have likely been infected in 2015; and 32.2% (172/534) likely acquired the infection between 2016 and 2017. A large proportion (79%) of the participants self-reported having been infected with HIV in Northern South Africa. Ninety-nine percent (529/534) of the participants initiated ART on a regimen of tenofovir, emtricitabine and efavirenz (Table 1). A total of 98% (523/534) of participants exited the study at 24 months. Fifty three percent (279/523) of participants who exited the study had defaulted ART, whereas 24% (126/523) transferred to different treatment centres. There were 21% (110/523) of participants who exited the study but stayed on ART and 2% (8/523) died.

**Table 1:** Demographic characteristics of the study cohort at HIV treatment initiation.

Characteristic	Overall, N = 534^1^	CD4 <500, N = 402^1^	CD4 >500, N = 83^1^	(Missing), N = 49^1^	p-value^2^
**Sex, n (%)**					0.002
*Female*	378 (71%)	272 (68%)	72 (87%)	34 (69%)	
*Male*	156 (29%)	130 (32%)	11 (13%)	15 (31%)	
**Age (in Years), Median (IQR)**	35 (IQR: 29–44)	36 (IQR: 29–44)	30 (IQR: 26–40)	36 (IQR: 27–48)	0.003
*Unknown*	1	1	0	0	
**Age ranges, n (%)**					0.39
*Less than or equal 20*	11 (2.1%)	7 (1.7%)	2 (2.4%)	2 (4.1%)	
*Between 21 and 40*	343 (64%)	257 (64%)	60 (72%)	26 (53%)	
*Between 41 and 60*	169 (32%)	129 (32%)	20 (24%)	20 (41%)	
*Greater than 60*	10 (1.9%)	8 (2.0%)	1 (1.2%)	1 (2.0%)	
*Unknown age*	1 (0.2%)	1 (0.2%)	0 (0%)	0 (0%)	
**Marital Status, n (%)**					0.10
Unknown status	3 (0.6%)	2 (0.5%)	1 (1.2%)	0 (0%)	
*Divorced*	9 (1.7%)	7 (1.7%)	1 (1.2%)	1 (2.0%)	
*Married*	164 (31%)	131 (33%)	16 (19%)	17 (35%)	
*Single*	346 (65%)	255 (63%)	63 (76%)	28 (57%)	
*Widowed*	12 (2.2%)	7 (1.7%)	2 (2.4%)	3 (6.1%)	
**Education, n (%)**					
*Unknown education level*	5 (0.9%)	3 (0.7%)	1 (1.2%)	1 (2.0%)	
*No formal education*	11 (2.1%)	10 (2.5%)	1 (1.2%)	0 (0%)	
*Primary school*	5 (0.9%)	5 (1.2%)	0 (0%)	0 (0%)	
*Some primary school*	107 (20%)	81 (20%)	15 (18%)	11 (22%)	
*Secondary school*	194 (36%)	143 (36%)	35 (42%)	16 (33%)	
*Some secondary school*	163 (31%)	123 (31%)	23 (28%)	17 (35%)	
*Post-secondary school*	49 (9.2%)	37 (9.2%)	8 (9.6%)	4 (8.2%)	
**Monthly income (ZAR), n (%)**					
*Unknown income*	23 (4.3%)	16 (4.0%)	5 (6.0%)	2 (4.1%)	
*Less than R3 000*	158 (30%)	125 (31%)	23 (28%)	10 (20%)	
*Greater than R10 000*	13 (2.4%)	11 (2.7%)	1 (1.2%)	1 (2.0%)	
*Not applicable (students and unemployed)*	265 (50%)	195 (49%)	45 (54%)	25 (51%)	
*R3 000 to R10 000*	75 (14%)	55 (14%)	9 (11%)	11 (22%)	
**Employment Status, n (%)**					0.63
*Unknown employment status*	6 (1.1%)	4 (1.0%)	1 (1.2%)	1 (2.0%)	
*Employed*	263 (49%)	203 (50%)	37 (45%)	23 (47%)	
*Unemployed*	265 (50%)	195 (49%)	45 (54%)	25 (51%)	
**Geographical area lived in when acquired HIV infection, n (%)**					
*Unknown*	67 (13%)	42 (10%)	12 (14%)	13 (27%)	
*Gauteng*	24 (4.5%)	19 (4.7%)	4 (4.8%)	1 (2.0%)	
*KwaZulu Natal*	4 (0.7%)	3 (0.7%)	1 (1.2%)	0 (0%)	
*Lesotho*	1 (0.2%)	1 (0.2%)	0 (0%)	0 (0%)	
*Limpopo*	420 (79%)	327 (81%)	60 (72%)	33 (67%)	
*Mpumalanga*	2 (0.4%)	1 (0.2%)	1 (1.2%)	0 (0%)	
*Northwest*	2 (0.4%)	1 (0.2%)	1 (1.2%)	0 (0%)	
*Zimbabwe*	14 (2.6%)	8 (2.0%)	4 (4.8%)	2 (4.1%)	
**Probable Year of HIV infection,** **n (%)**					0.063
*2002–2014*	163 (30.5%)	132 (32.8%)	22 (27%)	9 (18.4%)	
*2015*	191 (35.8%)	147 (36.6%)	26 (31%)	18 (36.7%)	
*2016–2017*	172 (32.2%)	117 (29.1%)	35 (42%)	20 (40.8%)	
*Unknown Year of infection*	8 (1.5%)	6 (1.5%)	0 (0%)	2 (4.1%)	
**WHO clinical diagnosed HIV-1 Stage, n (%)**					
*1*	422 (79%)	318 (79%)	76 (92%)	28 (57%)	
*2*	49 (9.2%)	46 (11%)	2 (2.4%)	1 (2.0%)	
*3*	11 (2.1%)	11 (2.7%)	0 (0%)	0 (0%)	
*4*	4 (0.7%)	4 (1.0%)	0 (0%)	0 (0%)	
*Unknown*	48 (9.0%)	23 (5.7%)	5 (6.0%)	20 (41%)	
**Tuberculosis infection, n (%)**					0.002
*Yes*	95 (18%)	85 (21%)	6 (7.2%)	4 (8.2%)	
*No*	439 (82%)	317 (79%)	77 (93%)	45 (92%)	
**Cryptococcus infection, n (%)**					0.65
*Yes*	7 (1.3%)	7 (1.7%)	0 (0%)	0 (0%)	
*No*	527 (99%)	395 (98%)	83 (100%)	49 (100%)	
**Herpes co-infection, n (%)**					0.45
*Yes*	6 (1.1%)	5 (1.2%)	0 (0%)	1 (2.0%)	
*No*	528 (99%)	397 (99%)	83 (100%)	48 (98%)	
**Bacterial infections, n (%)**					<0.001
*Yes*	72 (13%)	69 (17%)	3 (3.6%)	0 (0%)	
*No*	462 (87%)	333 (83%)	80 (96%)	49 (100%)	
**Fungal infection, n (%)**					0.77
*Yes*	6 (1.1%)	6 (1.5%)	0 (0%)	0 (0%)	
*No*	528 (99%)	396 (99%)	83 (100%)	49 (100%)	
**Non-communicable diseases (hypertension, diabetes, and asthma), n (%)**					0.12
*Yes*	11 (2.1%)	6 (1.5%)	4 (4.8%)	1 (2.0%)	
*No*	523 (98%)	396 (99%)	79 (95%)	48 (98%)	
**ARV regimen, n (%)**					>0.99
*ABC/3TC/EFV*	3 (0.6%)	3 (0.7%)	0 (0%)	0 (0%)	
*AZT/3TC/EFV*	2 (0.4%)	2 (0.5%)	0 (0%)	0 (0%)	
*TDF/FTC/EFV*	529 (99%)	397 (99%)	83 (100%)	49 (100%)	
**TB medication (RHZE and INH), n (%)**					0.004
*Yes*	79 (15%)	71 (18%)	6 (7.2%)	2 (4.1%)	
*No*	455 (85%)	331 (82%)	77 (93%)	47 (96%)	
**Acyclovir (Antiviral), n (%)**					>0.99
*Yes*	2 (0.4%)	2 (0.5%)	0 (0%)	0 (0%)	
*No*	532 (100%)	400 (100%)	83 (100%)	49 (100%)	
**Antibiotics (Augmentin, Amoxicillin, Ceftriaxone, cloxacillin, erythromycin, flagyl), n (%)**					0.56
*Yes*	8 (1.5%)	8 (2.0%)	0 (0%)	0 (0%)	
*No*	526 (99%)	394 (98%)	83 (100%)	49 (100%)	
**Medication for non-communicable diseases, n (%)**					0.14
*Yes*	5 (0.9%)	2 (0.5%)	2 (2.4%)	1 (2.0%)	
*No*	529 (99%)	400 (100%)	81 (98%)	48 (98%)	
**Bactrim, n (%)**					<0.001
*Yes*	90 (17%)	87 (22%)	3 (3.6%)	0 (0%)	
*No*	444 (83%)	315 (78%)	80 (96%)	49 (100%)	
**Vitamin and mineral supplements, n (%)**					0.002
*Yes*	190 (36%)	154 (38%)	30 (36%)	6 (12%)	
*No*	344 (64%)	248 (62%)	53 (64%)	43 (88%)	
**Flucozole (antifungal), n (%)**					0.77
*Yes*	6 (1.1%)	6 (1.5%)	0 (0%)	0 (0%)	
*No*	528 (99%)	396 (99%)	83 (100%)	49 (100%)	
**CD4 measurement pre-ART initiation (cells per μl blood), n (%)**					
*Less than 50*	51 (9.6%)	51 (13%)	0 (0%)	0 (0%)	
*50–100*	47 (8.8%)	47 (12%)	0 (0%)	0 (0%)	
*101–200*	86 (16%)	86 (21%)	0 (0%)	0 (0%)	
*201–350*	116 (22%)	116 (29%)	0 (0%)	0 (0%)	
*351–500*	102 (19%)	102 (25%)	0 (0%)	0 (0%)	
*501–1024*	83 (16%)	0 (0%)	83 (100%)	0 (0%)	
*Not measured*	49 (9.2%)	0 (0%)	0 (0%)	49 (100%)	
**Percentage CD4+ cells pre-ART initiation, n (%)**					
*Less than 5%*	50 (9.4%)	50 (12%)	0 (0%)	0 (0%)	
*5%– 10%*	69 (13%)	69 (17%)	0 (0%)	0 (0%)	
*11%– 20%*	161 (30%)	158 (39%)	3 (3.6%)	0 (0%)	
*21%– 30%*	130 (24%)	87 (22%)	43 (52%)	0 (0%)	
*31%– 40%*	56 (10%)	26 (6.5%)	30 (36%)	0 (0%)	
*41%– 50%*	3 (0.6%)	0 (0%)	3 (3.6%)	0 (0%)	
*Not measured*	65 (12%)	12 (3%)	4 (4.8%)	49 (100%)	
**Hemoglobin measurements pre-ART initiation (grams per decilitre), n (%)**					
*Less than 2*	5 (0.9%)	0 (0%)	0 (0%)	5 (10%)	
*2*.*1–8*	32 (6.0%)	30 (7.5%)	1 (1.2%)	1 (2.0%)	
*8–10*.*9*	139 (26%)	118 (29%)	18 (22%)	3 (6.1%)	
*11–11*.*9*	81 (15%)	62 (15%)	17 (20%)	2 (4.1%)	
*Greater than 12*	230 (43%)	175 (44%)	43 (52%)	12 (24%)	
*Not measured*	47 (8.8%)	17 (4.2%)	4 (4.8%)	26 (53%)	
**Viral load measurement pre-ART initiation (viral count per ml blood), n (%)**					
*Not detected– 20*	19 (3.6%)	8 (2.0%)	9 (11%)	2 (4.1%)	
*20–50*	5 (0.9%)	3 (0.7%)	1 (1.2%)	1 (2.0%)	
*51–1000*	45 (8.4%)	24 (6.0%)	18 (22%)	3 (6.1%)	
*1001–7283606*	381 (71%)	312 (78%)	44 (53%)	25 (51%)	
*Not measured*	84 (16%)	55 (14%)	11 (13%)	18 (37%)	

^1^median (IQR) for continuous variables; n (%) for categorical variables.

^2^Pearson’s Chi-squared test; Kruskal-Wallis rank sum test; Fisher’s exact test.

### Immunologic outcomes of participants at baseline and within 24 months of ART in the study, and associated factors

Results for CD4+ cell count were available for 485 of the eligible 534 participants prior to ART initiation. Error reading was obtained for CD4+ cell count measurements for 49 specimens. Repeated CD4+ T cell count testing was done as recommended by the manufacturer. However, no reading was obtained. Error reading could have been due to the presence of high concentrations of naturally occurring interfering agents within these specimens, diminished cell signal, or unexpected cell distribution in the sample. Of the available readings, 402 (75%) were LIA and 83 (16%) were EIA (Table 1). Sixty-eight percent of all LIA (272/402) and 87% of all EIA (72/83) were women, whereas 32% of all LIA (130/402) and 13% of all EIA (11/83) were men (p = 0.002). The median age for EIA was 30 years, compared to 36 years for LIA (p = 0.003).

**Fig 2:**
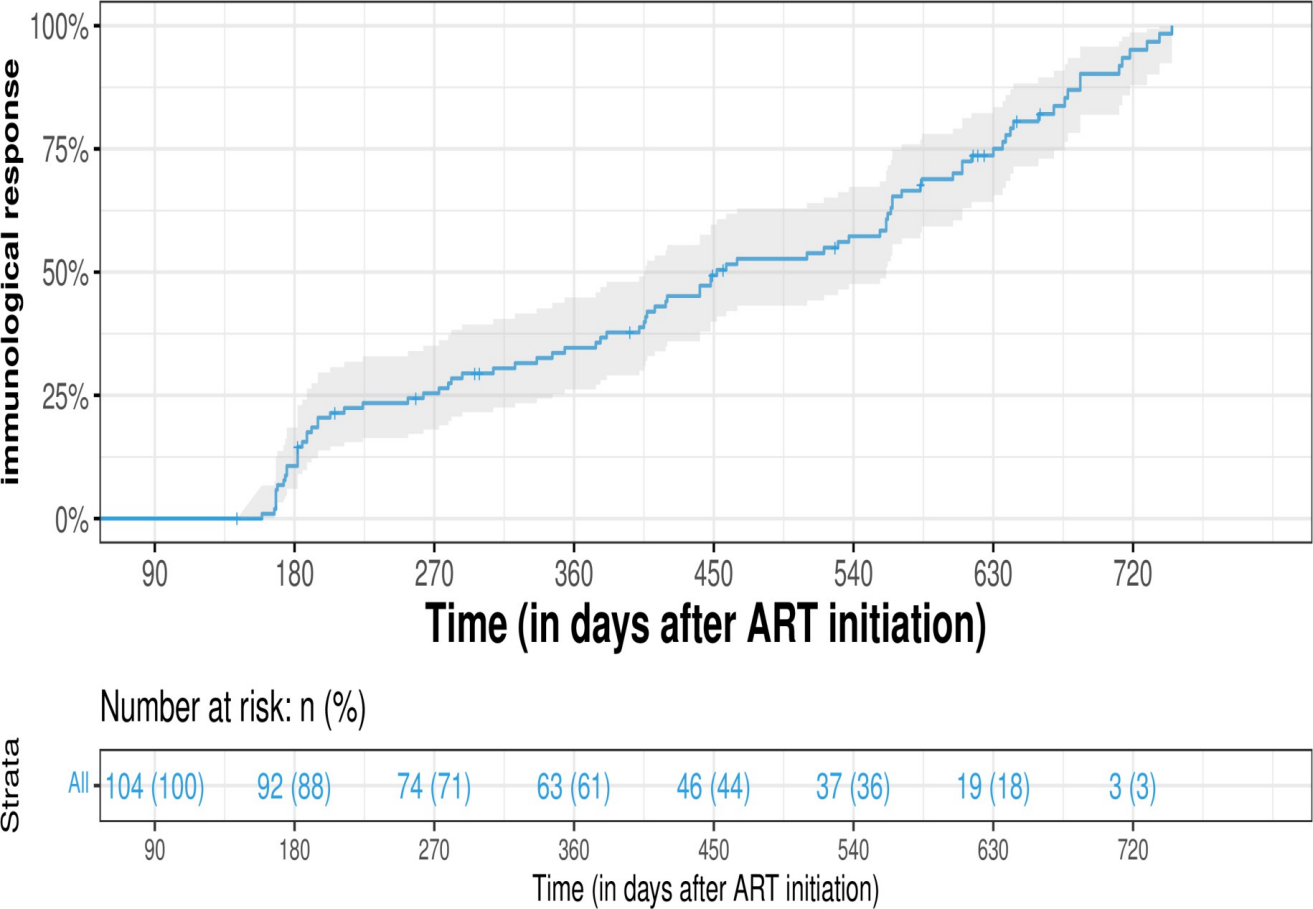
Kaplan-Meier estimate for immunological response among PLWH on ART over a 720-day (24 months) period after initiating ART.

A total of 104 participants on ART had CD4+ data for immunological response analysis. At 180 days (approximately 6 months) after ART, 11.5% (12/104) of participants had achieved a positive immunologic response. After 360 days (approximately 12 months on ART), 39.4% (41/104) had achieved a positive immunologic response. Ninety-eight percent (101/104) achieved positive immunological outcomes after 720 days (approximately 24 months) (Fig 2).

Multivariable cox proportional analysis was done to find associations between the outcome (immunological response) and the predictor variables. There was a statistically significant negative association between immunological response and individuals who were classified as WHO clinical stage 1 (AHR = 0.173; 95% CI: 0.0460–0.655; p = 0.0097) and clinical stage 3 (AHR = 0.123; 95% CI: 0.0155–0.977; p = 0.0475). There was also a highly significant negative association of immunologic response among individuals who had baseline CD4+ cell counts between 350 and 500 (AHR = 0.285; 95% CI: 0.1128–0.719; p<0.0079). There were however no significant associations between immunological response and baseline bacterial infections (AHR = 0.195; 95% CI: 0.0345–1.108; p = 0.0651) or TB infections (AHR = 0.537; 95% CI: 0.0722–3.989; p = 0.543).

### Virologic outcomes of participants at baseline and within 24 months of ART in the study, and associated factors

Seventy-one percent (381/534) of the participants had viral loads greater than 1000 copies/mL, while 4% (24/534) had a viral load less than 20 copies/mL, prior to treatment initiation. Quality viral load data was not available for 16% (84/534) of the participants prior to ART initiation (Table 1).

**Fig 3:**
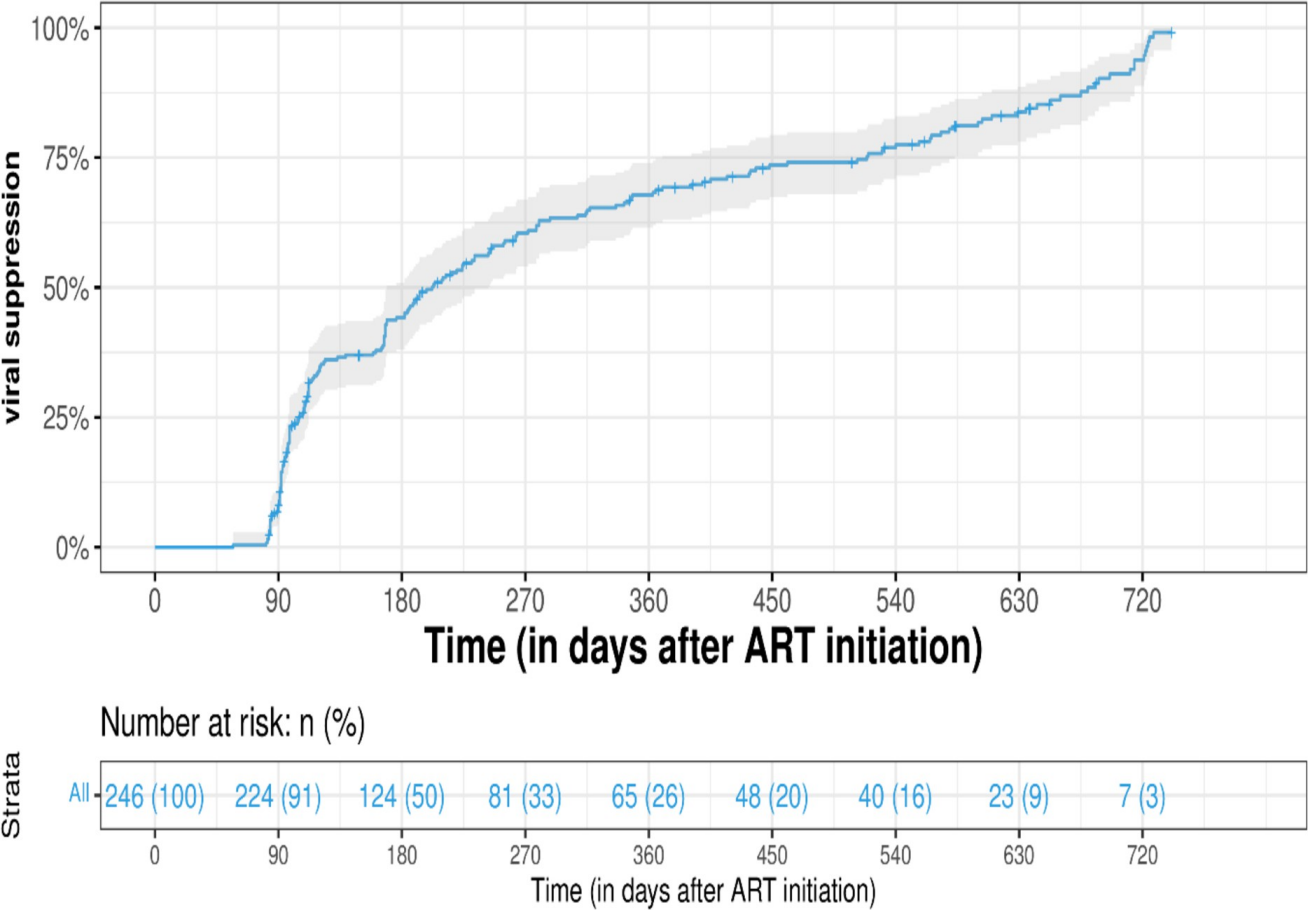
Kaplan-Meier estimate for PLWH reaching viral suppression over a 720 days (24 months) period after initiating ART. The grey shadow around the curve represents the confidence interval which was set at 95%.

For the Kaplan-Meier analysis, individuals who did not reach the primary treatment outcome at their last follow up were censored at their final follow up whereas the first follow up to reach the primary outcome was considered to have met the outcome. After 90 days (approximately 3 months), 9% (22/246) of participants achieved viral suppression. After 180 days (approximately 6 months) on ART, 50% (122/246) of participants achieved viral suppression; while 73.6% (181/246), achieved viral suppression after 360 days (approximately 12 months) on treatment. Ninety-seven percent (239/246) reached viral suppression after 720 days (approximately 24 months) of starting ART as shown in Fig 3.

There was a statistically significant difference in viral suppression between males and females. Fifty-two percent (91/175) of the females attained viral suppression after 180 days (approximately 6 months) of initiating ART compared to 43.7% (31/71) of the males over the same period (p = 0.021). Ninety-eight percent (172/175) of females attained viral suppression after 720 days (approximately 24 months) of ART to 94.7% (67/71) of males (p = 0.012).

When viral suppression was stratified with ART initiation based on CD4+ cell count, it was observed that EIAs were achieving viral suppression earlier compared to LIA, but this was not statistically significant (p = 0.33).

The multivariable cox proportional model shows the associations between the primary outcome (viral suppression) and the predictor variables, namely sex, age, occupation, monthly income, marital status, TB infection, bacterial infection, hemoglobin count, ART initiation and WHO clinical HIV stage. The male sex and hemoglobin count (>12g/dl of blood) were independent predictors of viral suppression. There was a statistically significant negative association between viral suppression and males (AHR = 0.624; 95% CI: 0.4124–0.944; p = 0.0257). In contrast, there was a statistically significant positive association between viral suppression and hemoglobin >12g/dL (AHR = 2.307; 95% CI: 1.6067–3.312; p<0.001). There was a non-significant positive association between viral suppression and people with TB infection prior to initiating ART (AHR = 1.054; 95% CI: 0.6901–1.611; p = 0.8063). Also, there was a non-significant negative association between viral suppression and bacterial infections prior to ART initiation ART (AHR = 0.611; 95% CI: 0.3533–1.057; p = 0.0779).

### HIV-1 drug resistance outcomes of participants on ART in the study

**Fig 4:**
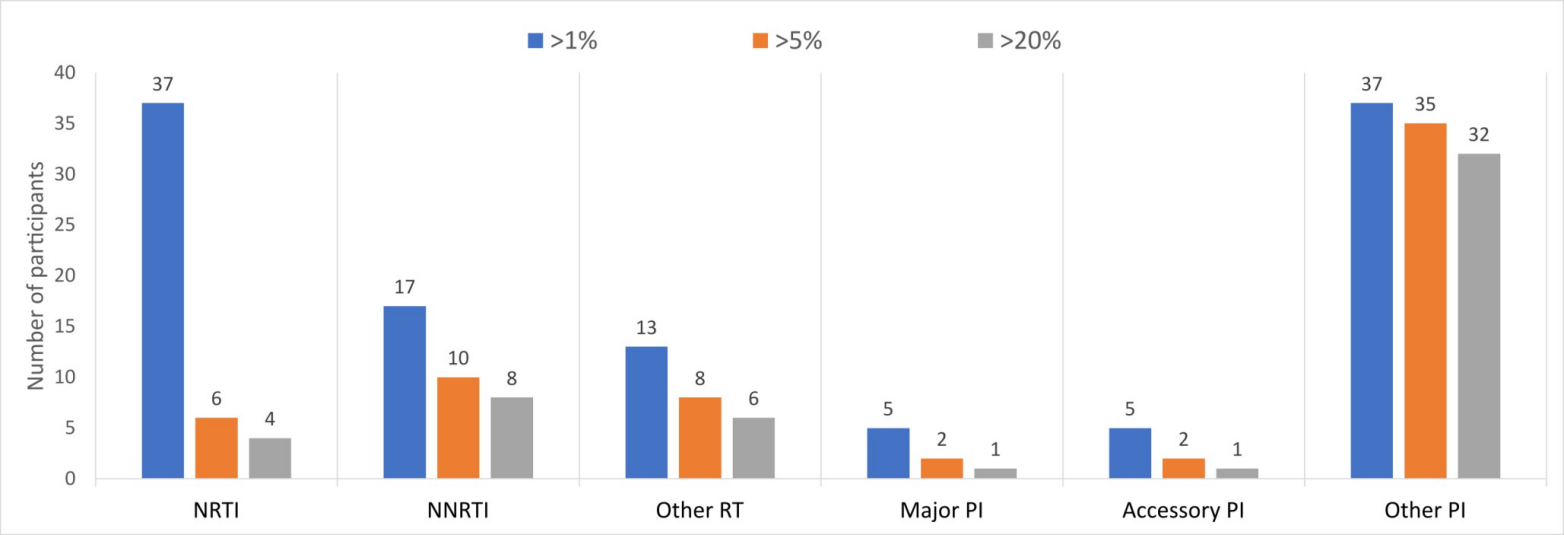
Total number of participants with DRM present at all 4 timepoints categorized by ARV drug classes.

A total of 203 baseline samples which had follow up samples collected at either 3, 6, 9 or 12 months after treatment initiation were considered for drug resistance analysis. Of the total considered, 114 samples were successfully analysed. A total of 114 samples were used to infer HIV-1 drug resistance, with 3 consensus sequences generated per sample at >20% threshold (majority) and >5%, >1% thresholds (minority) to account for minor variants. Of these, overall 45.6% (52/114) had at least one DRM detected at >20% threshold and increased to 55.3% (63/114) when minor variants were accounted for at >5% and >1% thresholds. The NRTIs were the most prevalent at >1% threshold with 32.5% (37/114), while other PIs were most prevalent in all the thresholds (Fig 4).

**Fig 5:**
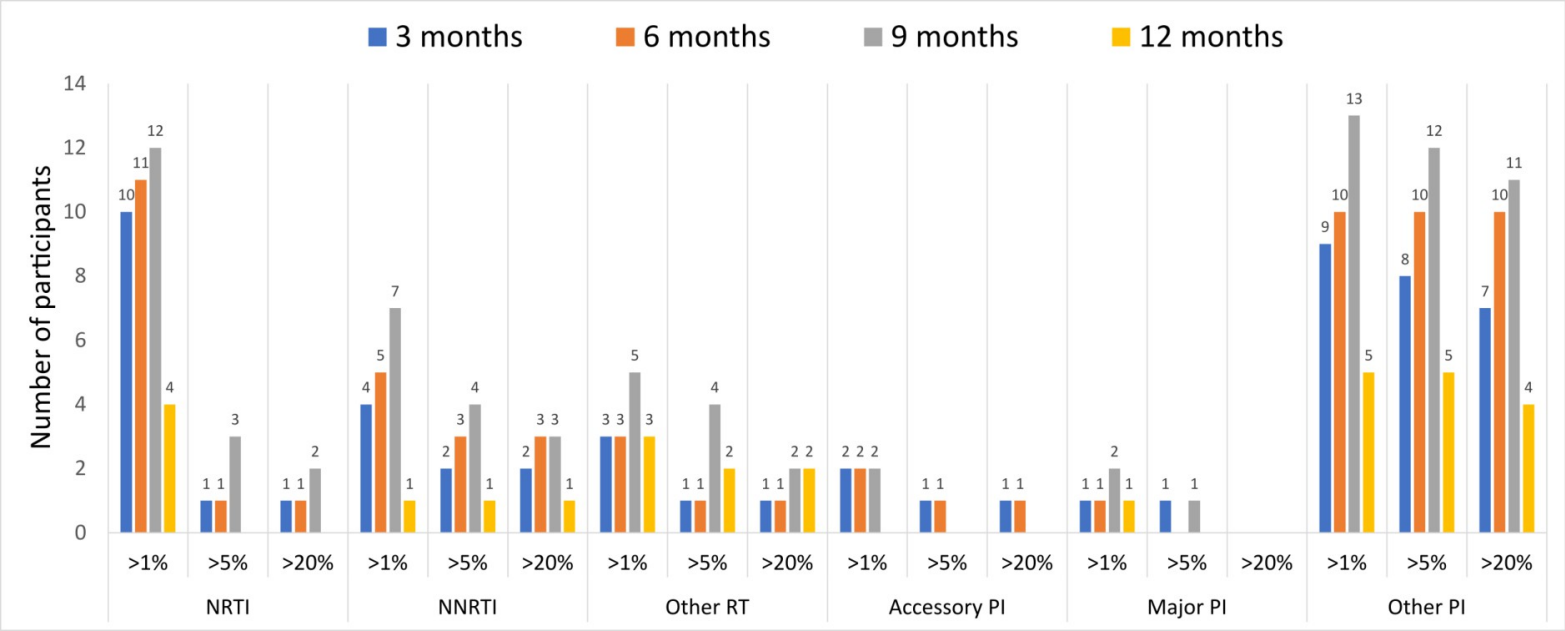
Number of participants with DRM detected at >1%, >5% and >20% viral thresholds at each time-point categorized by ARV drug classes.

The prevalence NRTI DRM at >20% threshold at 3 months was 3.4% (1/29), at 6 months was 2.7% (1/37), at 9 months was 6.1% (2/33), and 0% (0/15) at 12 months. The NRTI prevalence increased at 9 months by 9.1% (3/33) when minor variants were accounted for at >5% and 36.4% (12/33) at >1% thresholds. The NNRTI DRM prevalence at >20% threshold was 6.9% (2/29), 8.1% (3/37), 9.1% (3/33) and 6.7% (1/15) at 3 months, 6 months, 9 months, and 12 months timepoints respectively. This prevalence increased at 9 months by 12.1% (4/33) at >5% threshold and 21.2% (7/33) at >1% when minor variants were accounted for. There were no major PI DRMs observed at any of the timepoints at >20% viral threshold. However, the PI DRM prevalence at >5% threshold was 3.4% (1/29) and 3% (1/33) at 3 months and 9 months timepoints, with no PI DRM at 6 and 12 months timepoints (Fig 5). The most prevalent NRTI DRM were M184I/V and K70E/R and T215I, while E138A/G/K, K103N, P225H and K101E were the most NNRTIs. The PI mutations most prevalent were K20R, M46I and V82A.

### HIV-1 drug resistance outcomes of participants on ART in the study and viral load correlation

**Fig 6:**
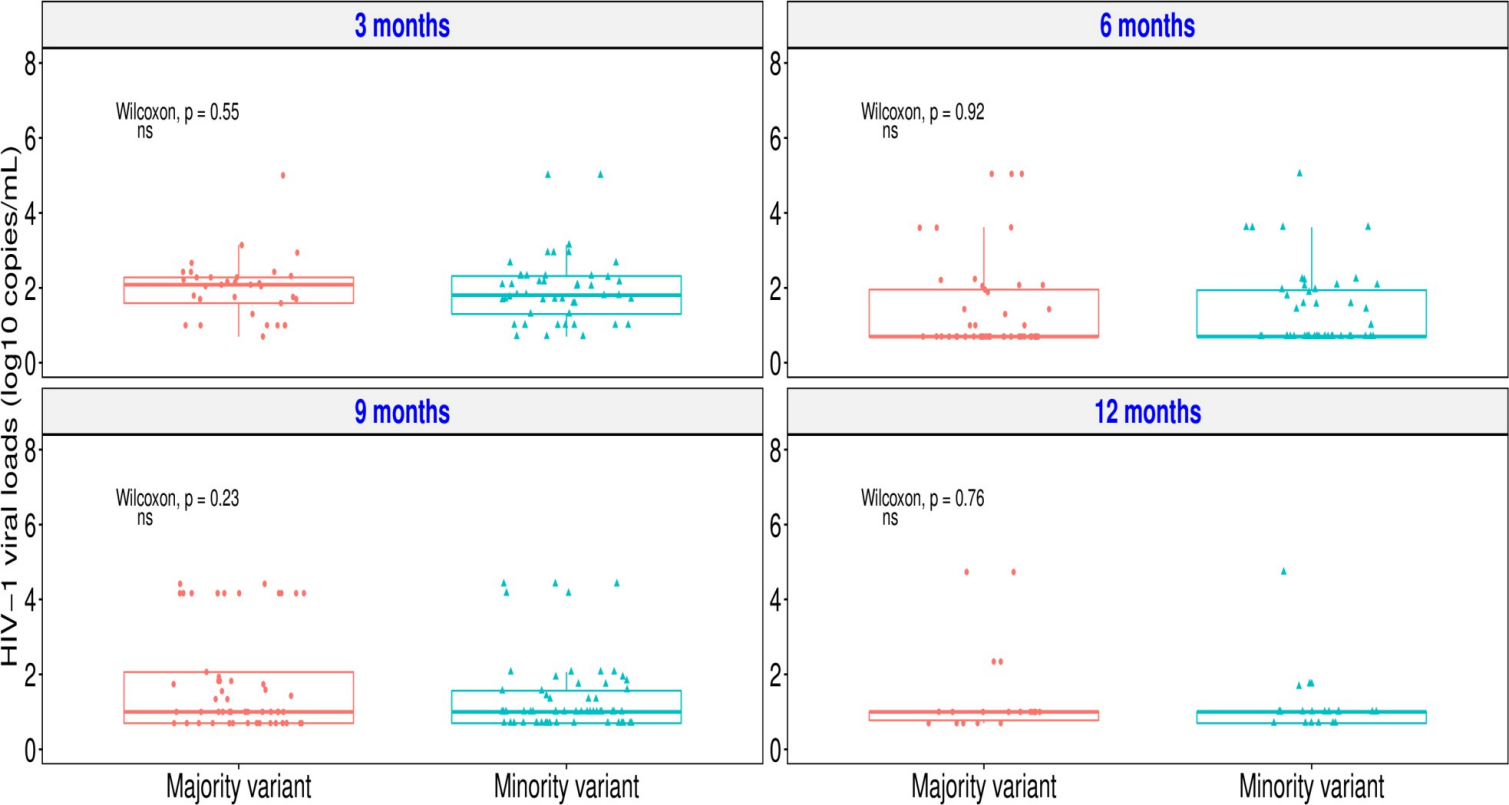
Relationship between viral load and HIV-1 drug resistance variants at the different timepoints post ART initiation among individuals on ART. Ns = no significance.

Drug resistance mutations were categorized as those detected at a majority threshold of >20% and <20% to represent minority threshold of >5% and >1%. At the three months timepoint, there was a difference in median viral load (measured in Log_10_ HIV) among those with majority variants (median = 2.08; IQR: 1.59–2.28) and those with minority variants (median = 1.81; IQR: 1.30–2.31). This difference was not statistically significant (p value = 0.55). At the six months timepoint, there was no difference in median viral load among majority variants (median = 0.7; IQR: 0.70–1.95) and minority variants (median = 0.7; IQR: 0.70–1.94) and this lack of difference was not statistically significant (p value = 0.92). At the nine months and 12 months timepoint, there was no difference in majority variants (median at 9 months = 1; IQR: 0.70–2.06; median at 12 months = 1; IQR: 0.78–1.00) and minority variants (median at 9 months = 1; IQR: 0.70–1.56; median at 12 months = 1; IQR: 0.70–1.00). The p value at nine months was 0.23 and at 12 months was 0.76. There was a drop in viral load for both variants at 3 month time-point from 2 log_10_ copies per ml at 3 months to 1 log_10_ copies per ml at 6 months timepoint. (Fig 6). This decline occurred at an identical amount among minority variants and majority variants.

### HIV phylogenetic analysis

**Fig 7:**
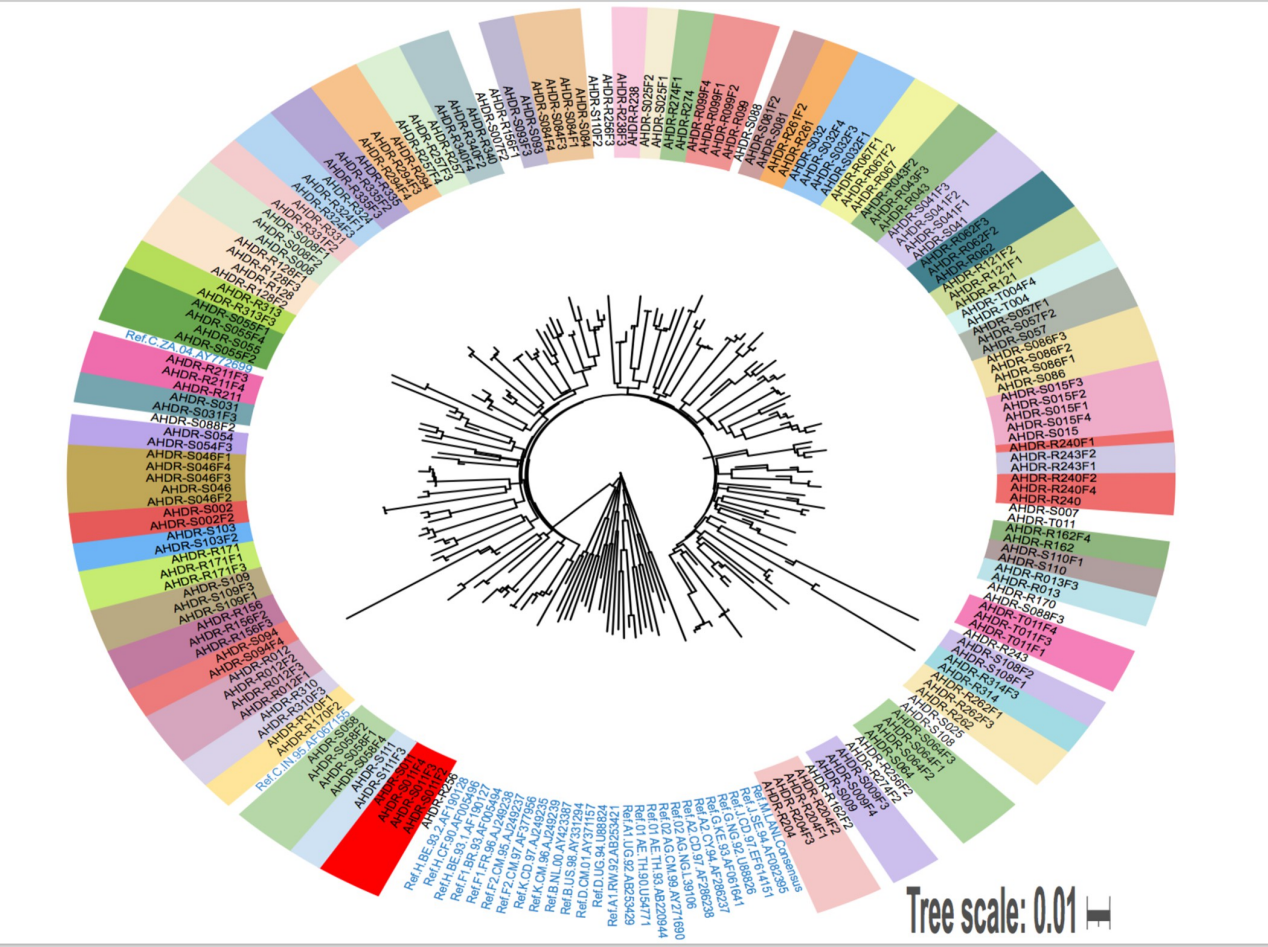
Phylogenetic tree of 144 samples showing clustering with HIV-1 subtype C reference sequences. Maximum likelihood tree generated using Tamura-Nei model bootstrapped to 1000 replicates and annotated using iTOL. Test sequences for each participant at the different timepoints are coded as AHDR and a number.

All 114 (100%) of the consensus NGS sequences clustered and intermingled with HIV-1 subtype C reference sequences from different geographic locations (Fig 7).

## Discussion and conclussion

The UNAIDS goal of 95-95-95 by 2030 is expected to significantly reduce HIV transmission and bring an end to the epidemic. This observational study investigated drug resistance, immunologic and virologic treatment outcomes among HIV-1 positive individuals initiating ART in the ‘Test and Treat’ program in Northern South Africa. The major success of the UTT program and achieving 95% sustained viral suppression depends on a myriad of factors with the most important factor being retention in care of PLHIV [15,16]. However, this has been challenged by loss to follow-up which is a major public concern [17]. In this study, it was observed that almost all the participants (98%) exited the study at 24 months. Measures such as follow up calls after missed appointment dates, organizing alternative convenient times and locations for follow-ups, were taken to maintain participant retention within the study. However, it did not contribute to a lasting increase in retention. Of the 98% participants who exited the the study, 52.3% (279/534) were lost to follow up, 23.6% (126/534) were treatment center transfers, 20.6% (110/534) exited study but remained on ART whereas 1.5% (8/534) had died.

High rates of lost to follow up have been reported in other UTT studies from different parts of South Africa [15,18,19]. A study by [20 and colleagues] particularly evaluated lost to follow-up in a pre-UTT group and post-UTT group, and lost to follow-up was 1.5 times higher among the post-UTT group compared to the pre-UTT group [20]. Increased rates of lost to follow-up in the UTT program may be attributed to the fact that a majority of individuals initiating first-line therapy may often be in better health, subsequently not perceiving a direct benefit to treatment, which may deter individuals from consistent engagement in HIV treatment programmes. Groups shown to be at risk of lost to follow up are male and young [20]. These groups generally have poor engagement with healthcare workers and have irregular lifestyle routines such as constantly moving around for work and also engaging in high-risk sexual behaviors. Loss to follow-up leads to increased treatment failure, viral rebound, and drug resistance [17].

In this study, 71% had baseline viral loads greater than 1000 copies/mL and 75% were late ART initiators prior to starting ART. This indicated that most people initiating ART were either not being diagnosed early enough or were diagnosed early but initiated treatment late. A smaller portion of these individuals (18%) had tuberculosis infections that could have delayed initiating ART. Other studies have also shown that delays to ART initiation could be due to late diagnosis or other opportunistic infections [21,22]. It is also possible that treatment centres re-initiated ART for people who defaulted without disclosure when UTT started in September 2016.

Immunologic response was achieved by 11.5% of the participants after 6 months and by 39.4% after 12 months of ART initiation. It has been shown that immunological response is generally slow and requires a long time on ART for increases to be observed. A study that supports our findings showed that among United States of America and Canadian PLWH followed up for a year after initiating ART, 56% (127/228) of the participants had a CD4+ cell count increase of fewer than 50 cells/μL after 1 year of ART [23]. However, our findings are contradicted by a study of a Mongolian study that showed that among 180 PLWH on ART with a baseline median CD4+ cell count of 327.9 cells/μL, immunological response (that is an increase of baseline CD4+ cell count by 50 cells/μL) was seen in 62.7% of the participant after 3 months of starting ART, 80.7% of participants after 12 months and 89.2% after 36 months [24,25]. A study by [26] evaluated the impact of UTT program on mean CD4 count in rural settings in South Africa. The key findings indicate that although UTT led to an immediate increase in the CD4 counts at ART initiation, the long-term effects were modest. It was also observed that men had initiated ART at lower CD4 counts than women [26]. Therefore, more efforts are needed to increase the initiation of ART early in those living with HIV, particularly men.

Nine percent achieved viral suppression after 3 months of initiating ART. Fifty percent attained viral suppression after 6 months of initiating ART, with 73.6% reaching viral suppression after 12 months of starting ART and 97.2% after 24 months. Studies done in the Western Cape, KwaZulu Natal, Limpopo, and Mpumalanga provinces of South Africa also observed almost similar patterns of viral suppression [27–29]. [29 and colleagues] also found that viral suppression was negatively associated with the male gender, a similar observation to this study. This association has been observed in another South African study [19]. A cause for the association between men and poor virological outcome is likely due to sub-optimal adherence to ART, fear of the stigma of being seen as weak men by others due to having HIV and poverty [30–32].

Drug resistance is also a major setback in achieving virologic and immunologic outcomes. With the application of next-generation sequencing, we observed that participants had DRM as early as 3 months into treatment. The most prevalent DRM were the PI minor accessory resistance mutations; and NRTI resistance mutations respectively. The PI minor accessory resistance mutations with the highest frequency at the >20% threshold were K20R, L10I, T74S and V82I. These mutations are polymorphic and by themselves do not cause PI resistance. However, they have been shown to reduce susceptibility to PIs and in the presence of a major mutation, can result in cross-resistance. The prevalent DRMs against NRTIs were M184V and K65R, which are commonly prevalent among people with HIV-1 subtype C. The mutation M184V causes high-level resistance to tenofovir and emtricitabine, while K65R causes high-level resistance to abacavir and tenofovir (three recommended and widely used NRTIs). These mutations could pose challenges to people who switched to a TLD regimen as strains harbouring these DRMs against NRTIs could mean the regimen is effectively dolutegravir monotherapy [33].

The observation of participants harbouring DRMs as early as three-month post-ART initiation could indicate ongoing viral replication during ART, leading to the selection of strains under drug pressure and archiving of these strains during ART. This replication is possible due to suboptimal concentrations of the drug throughout the body; and this could be a result of poor drug penetration or due to suboptimal adherence to ART [34]. In our study, the NRTI prevalence ranged from 6.9% at 3 months, 8.1% (3/37) at 6 months, 21.2% (7/33) at 9 months, and none at 12 post treatment initiation. These were below the NRTI prevalence ranges from the national surveys [35]. The NNRTI prevalence ranged from 20.7% (6/29) at the 3 months, 24.3% (9/37) at 6 months, 27.3% (9/33) at 9 months, and 13.3% (2/15) at 12 months post treatment initiation. Our results were below the national survey prevalence [35]. This is likely because the national surveys only considered NRTI and NNRTI prevalences for people experiencing virological failure.

Increased uptake of ART in the setting of UTT could potentially increase the prevalence of drug resistance, especially in settings with limited access to routine viral load monitoring and drug resistance testing. Few studies have evaluated whether the provision of UTT is associated with an increase in drug resistance [6,36]. A study by [37] evaluated the impact of UTT on HIV drug resistance on seroconverters (infected <1 year), non seroconverters (infected >1 year) and individuals with an unknown duration of infection. The study found no association between UTT and increased drug resistance in the cohort. However, higher rates of drug resistance and multi-class resistance were observed in non-seroconverters compared to seroconverters, with NNRTI and NRTI the most frequent [37]. The application of resistance monitoring, the use of drugs with high genetic barriers to resistance and increased support for ART adherence may help reduce resistance in the UTT settings.

In conclusion, within a prospective, observational setting, a larger proportion of people who entered treatment at the start of UTT in Northern South Africa had a positive virologic and immunologic outcome, with more women achieving viral suppression compared to men. We have also shown that there was a variable DR prevalence within the first year of ART among people entering UTT. Some of the factors that could be contributing to the observed drug resistance could be sub-optimal adherence or/and low-level viral replication, which could be drivers of the continual evolution and selection of drug-resistant viruses. Future studies of viral evolution during low-level viral replication could elucidate the impact it could have on the current treatment regimen. Also, studies should investigate specific barriers to men achieving improved ART outcomes.
